# Cofilin Regulates Nuclear Architecture through a Myosin-II Dependent Mechanotransduction Module

**DOI:** 10.1038/srep40953

**Published:** 2017-01-19

**Authors:** O’Neil Wiggan, Bryce Schroder, Diego Krapf, James R. Bamburg, Jennifer G. DeLuca

**Affiliations:** 1 Department of Biochemistry and Molecular Biology, Colorado State University, Fort Collins, CO, 80523, USA; 2 School of Biomedical Engineering, Colorado State University, Fort Collins, CO, 80523, USA; 3 Department of Electrical and Computer Engineering, Colorado State University, Fort Collins, CO, 80523, USA; 4 Molecular Cellular and Integrative Neuroscience, Colorado State University, Fort Collins, CO 80523, USA

## Abstract

Structural features of the nucleus including shape, size and deformability impact its function affecting normal cellular processes such as cell differentiation and pathological conditions such as tumor cell migration. Despite the fact that abnormal nuclear morphology has long been a defining characteristic for diseases such as cancer relatively little is known about the mechanisms that control normal nuclear architecture. Mounting evidence suggests close coupling between F-actin cytoskeletal organization and nuclear morphology however, mechanisms regulating this coupling are lacking. Here we identify that Cofilin/ADF-family F-actin remodeling proteins are essential for normal nuclear structure in different cell types. siRNA mediated silencing of Cofilin/ADF provokes striking nuclear defects including aberrant shapes, nuclear lamina disruption and reductions to peripheral heterochromatin. We provide evidence that these anomalies are primarily due to Rho kinase (ROCK) controlled excessive contractile myosin-II activity and not to elevated F-actin polymerization. Furthermore, we demonstrate a requirement for nuclear envelope LINC (linker of nucleoskeleton and cytoskeleton) complex proteins together with lamin A/C for nuclear aberrations induced by Cofilin/ADF loss. Our study elucidates a pivotal regulatory mechanism responsible for normal nuclear structure and which is expected to fundamentally influence nuclear function.

Interphase intranuclear spatial organization is non-random^1^ and nuclear structural features including shape, size and deformability impact nuclear function affecting processes that are both normal, such as cell differentiation, and pathological such as tumor cell migration^2^,^3^. Despite the fact that abnormal nuclear morphology has long been a defining characteristic for diseases such as cancer^4^ little is known about the mechanisms that dictate the stereotypic spherical to ellipsoid morphology in most normal tissues and the aberrant nuclear morphologies of different diseases.

Structural integrity of the nucleus is dependent on properties of the nuclear lamina, consisting of A- and B-type lamin intermediate filaments^5^, and nuclear envelope proteins comprising the LINC (linker of nucleoskeleton and cytoskeleton) complex^6^. Mutations to these nuclear components result in dysmorphic nuclei and are associated with diverse human diseases collectively termed nuclear envelopathies^7^. The mechanisms contributing to pathology in these diseases are poorly understood but may involve perturbed gene regulation, altered cell mechanics and impaired force transduction between the nucleus and the cytoskeleton^8^,^9^,^10^,^11^. Organization of the cytoplasmic cytoskeleton network clearly influences nuclear morphology, largely due to mechanical coupling of the cytoskeleton to the nucleus via the LINC complex^12^,^13^,^14^,^15^,^16^,^17^,^18^. Still, the factors that regulate the balance of mechanical forces between the cytoskeleton and nucleus are poorly understood.

Cofilin-1 and ADF, products of separate genes, are best known for their ability to induce F-actin disassembly via filament severing or depolymerization^19^,^20^. We recently showed that proteins of the cofilin/ADF-family negatively regulate myosin-II activity through competitive inhibition for binding to F-actin^21^. We hypothesized that in addition to their well-known roles in regulating F-actin turnover, the cofilin/ADF proteins are important for the control of intracellular tensional homeostasis through modulation of actomyosin assembly. In further tests of this hypothesis we identify crucial roles for cofilin/ADF in control of cytoskeletal force modulation for maintenance of normal nuclear architecture. Our results support and extend to additional cell types the generality of the findings of Kanellos *et al*.^22^, which were published during preparation of our manuscript. Both studies highlight an importance for deregulated myosin-II contractile activity, coupled to mechanotransduction by nuclear envelope LINC complex proteins, in the genesis of nuclear structural deficits following loss of cofilin/ADF. In addition, our results provide insights as to how intracellular mechanical forces may act to influence nuclear morphology and chromatin organization.

## Results

### ADF/cofilin silencing induces abnormal nuclear morphologies

**Figure 1:**
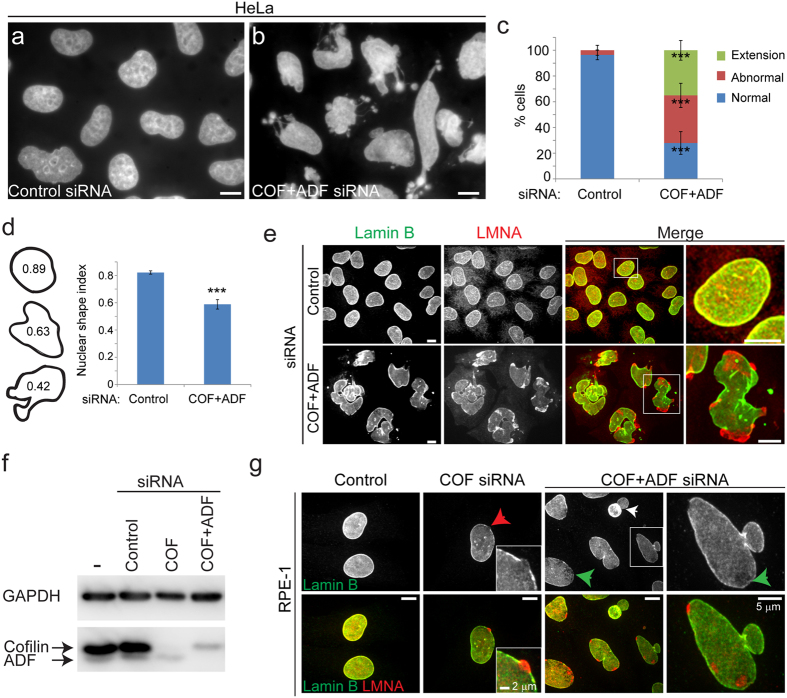
Cofilin/ADF depletion results in nuclear structural defects. Nuclear DAPI staining of control (**a**) and COF + ADF (**b**) siRNA treated HeLa cells fixed at 72 h post treatment. (**c**) Quantification of nuclear morphological defects. Abnormal includes the presence of multiple lobules, two or more invaginations, herniations and nuclei with clear deviation from a smooth spherical morphology but without an extension. Values are mean ± SD (n > 1400 cells/treatment), from at least 3 experiments. (**d**) Quantification of nuclear shape (circularity) index calculated as 4πArea/Perimeter^2^. Shape index values range from 0 (shape approaching that of a line) to 1 (a circle). Values are mean ± SD (n > 700/treatment). Tracings of exemplary nuclei and corresponding shape index are depicted. (**e**) Immunofluorescence labelling of lamin B and lamins A/C (LMNA) in HeLa cells. Boxed region in first merge column is magnified in second merge column. (**f**) Representative blot of cofilin/ADF, and GAPDH (loading control) at 72 h post siRNA treatments of RPE-1 cells. (**g**) Immunofluorescence labelling of lamin B and LMNA in RPE-1 cells. Red arrow depicts region of LMNA herniation at a site of disrupted lamin B, magnified in inset. Green arrows highlight abnormal porous lamin B staining and white arrow shows dumbbell shaped nucleus. Boxed region is magnified in last column. Bars, 10 μm. *** p ≤ 0.001, Welch’s t-test.

**Figure 2:**
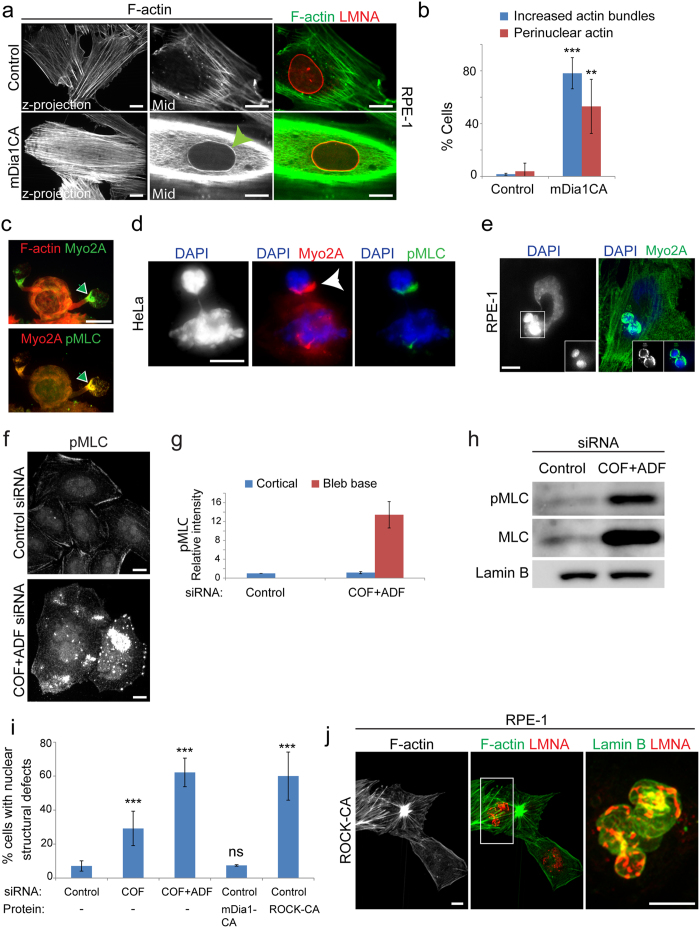
Nuclear structural defects following Cof/ADF depletion correlate specifically with excessive actomyosin assembly. (**a**) Confocal immunofluorescence images of control siRNA treated RPE-1 cells with or without expression of constitutively active mDia1 (mDiaCA), labeled for F-actin and LMNA. Second and third columns are single confocal sections at mid nuclear height. Green arrowhead shows perinuclear F-actin accumulation. (**b**) Quantification of RPE-1 cells with irregular increased numbers of F-actin bundles or the presence of a perinuclear F-actin ring as illustrated in (**a**). Values are mean ± SD, n > 450/treatment. **p ≤ 0.01, ***p ≤ 0.001, Welch’s t-test (**c**,**d**,**e** and **f**) Abnormal accumulation of myosin-2A and active myosin (p-MLC) at the base of blebs (**c**, green arrowhead and **f**) and around nuclear extensions (**d**, white arrowhead) in Cof/ADF siRNA treated HeLa cells (**c**,**d** and **f**) and RPE-1 cells (**e**). Images are of confocal z-projections. Boxed region (**e**), shown in insets, depicts a single apical z-plane. (**g**) Quantification of relative pMLC fluorescence intensity to non-bleb or plasma membrane bleb cortical sites. Values are mean ± SD, n > 30 cells/treatment. (**h**) Representative MLC immunoblots with lamin B as a loading control. (**i**) Quantification of nuclear structural defects in RPE-1 cells siRNA treated along with ectopic expression of proteins as indicated. Values are mean ± SD, ROCK-CA n = 94 and n > 270 for all other treatments. *p < 0.05, **p ≤ 0.01, ***p ≤ 0.001, ns, not significant, one-way ANOVA and Dunnett’s post hoc test, relative to control. (**j**) Immunofluorescence labeling of F-actin and lamins in ROCK-CA expressing RPE-1 cells. Boxed region is magnified in last panel. Bars, 10 μm.

Silencing of both Cofilin-1 and ADF (COF + ADF, Cof/ADF or C/A) using multiple siRNAs, previously shown to efficiently deplete both proteins^21^, resulted in severe nuclear structural defects (Fig. 1). Of control HeLa cells, 96% exhibited a stereotypic spherical nuclear morphology in contrast to Cof/ADF depleted cells where over 70% of cells displayed abnormalities classified in DAPI staining by the presence of multiple lobules and or invaginations, herniations or the presence of finger-like protrusions with bulbous ends (Fig. 1a–c). Nuclear dysmorphology was also clearly evident by a decrease in the circularity (nuclear shape index) of Cof/ADF silenced cells relative to controls (Fig. 1d, p = 0.0003, Student’s t-test). Cof/ADF silencing disrupted nuclear lamina organization such that the lamin B labeled nuclear lamina became porous and discontinuous in its normal overlapping co-localization with lamins A/C (products of a single gene, hereafter referred to as LMNA), at the nuclear periphery (Fig. 1e,g). Nuclear architectural defects following Cof/ADF depletion were not restricted to HeLa cells and were observed in multiple cell types including U2OS cells (not shown) and non-transformed RPE-1 cells (Figs 1f,g, 2e and 2i for quantification of lamin structural deficits in RPE cells). Defects resulting from depletion of cofilin alone were generally less severe both quantitatively and in quality relative to those from depletion of both cofilin and ADF, consistent with previous reports of functional redundancy^21^,^23^.

### Mechanisms contributing to nuclear abnormalities in cofilin/ADF silenced cells

By what mechanism does loss of Cof/ADF bestow the dramatic nuclear structural defects we observe? Prevailing models of Cof/ADF function would suggest increased F-actin assembly as the most likely prospect. We found this possibility improbable since induction of strong F-actin polymerization in RPE-1 cells, through expression of a constitutively active F-actin nucleator, the formin mDia1 (mDia1CA), did not significantly alter nuclear structure (Fig. 2a,b,i). This was so despite the fact that mDia1CA expression robustly stimulated increased assembly of thin F-actin stress fibers in the majority of cells (Fig. 2a,b). Furthermore, expression of active mDia1 induced strong F-actin assembly circumferentially and in close juxtaposition to the nucleus in over 50% of cells, with negligible impact to nuclear morphology (Fig. 2a,b,i). These data imply that induced F-actin accumulation, at least that induced by formin-family nucleators, is not by itself sufficient to induce aberrant nuclear morphology.

Many dynamic cellular operations rely on assemblies of both F-actin and the molecular motor myosin-II (actomyosin). Myosin-II activation and actomyosin assembly are stimulated by the activity of kinases such Rho kinase (ROCK) which mediate phosphorylation of the regulatory light chain subunit of myosin-II^24^. Silencing of Cof/ADF in HeLa cells resulted in a mean 13-fold increase in the accumulation of active myosin-II, marked by phosphorylated myosin light chain (p-MLC), to cortical sites at the base of previously characterized apical persistent non-apoptotic membrane bleb protrusions, which result from excessive myosin-II contractile activity (Fig. 2c,d,f,g and ref. 21). Increased myosin light chain phosphorylation was coupled to its increased expression in Cof/ADF depleted HeLa cells (Fig. 2h). Significantly, active myosin-II accumulated in a collar around bulbous nuclear extensions suggesting a role for myosin-II activity in formation of these structures. Unlike HeLa cells, few Cof/ADF depleted RPE-1 cells displayed prominent blebbing (<5%), however, amongst this population of RPE-1 cells, apical nuclear extensions associated with aberrant myosin accumulation were also observed (Fig. 2e).

We examined whether induced myosin-II activation, through expression of constitutively active Rho kinase (ROCK-CA) could alter nuclear morphology as observed in Cof/ADF depleted cells. In contrast to mDia1CA, expression of ROCK-CA induced nuclear structural defects including dysmorphologies and perturbations to the organization of the nuclear lamina in 60% of cells, similarly to COF + ADF depletion (Fig. 2i,j). These results are consistent with a model whereby excessive myosin-II contractile forces are at the crux of nuclear disorganization in Cof/ADF depleted cells. Together, the data here also highlight an important concept, that increased F-actin assembly does not obligate increased contractile actomyosin assembly nor are the two processes functionally synonymous. Our results thus suggested specific requirements for contractile myosin activity in nuclear morphology regulation, as opposed, for instance, to mere F-actin patterning such as through a scaffolding mechanism.

**Figure 3:**
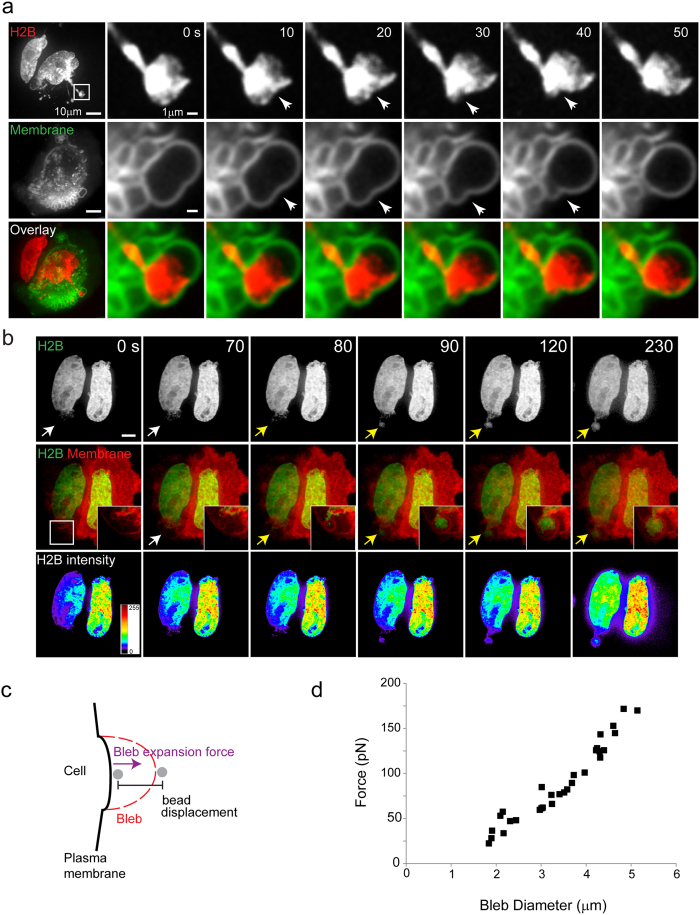
Mechanical coupling of nuclear extensions with plasma membrane blebs and bleb expansion force measurements. (**a**) Confocal z-projection images (first column) and time-lapse series (single confocal sections) of corresponding dynamic shape changes (arrows) to a nuclear extension (labeled by RFP-H2B) and plasma membrane bleb (labeled by GFP-CAAX) in a COF + ADF siRNA depleted HeLa cell. Boxed region (H2B, first panel) is magnified in the time-lapse series. (**b**) Time-lapse series of live COF + ADF depleted HeLa cells with nuclei (H2B, green) and membranes (red) depicting the location prior to (white arrows) and during (yellow arrows) formation of a plasma membrane bleb and nuclear extension. Insets are magnified regions marked by arrows and boxed region (first middle panel). Bars, 10 μm. (**c**) Schematic of the experimental setup for bleb expansion force measurements using optical tweezers as described in Methods. (**d**) Force measured with optical tweezers during bleb expansion as a function of bleb size.

Live cell imaging of Cof/ADF depleted HeLa cells showed that nuclear extensions associated with apical plasma membrane blebs cells were dynamically shaped by their apparent coupling to the plasma membrane protrusions in which they were embedded (Fig. 3a). For example, contractions to the bleb membrane over a period of 30 s resulted in simultaneous indentation to the portion of the nucleus embedded in the bleb (Fig. 3a, arrows). These observations insinuated that nuclei in Cof/ADF depleted cells became highly compliant and mechanically coupled to the apical plasma membrane. In further support of this mechanical coupling, we observed that nuclear extensions frequently arose in conjunction with rapid expansion of a membrane bleb, over a period of seconds (Fig. 3b, yellow arrows). Intensity maps of GFP-H2B revealed dynamic redistribution of chromatin during the period of formation of this nuclear extension (Fig. 3b, bottom row). Furthermore, as illustrated in Supplementary Fig. S1, a force exerted on the nucleus resulted in localized upward extension of the nucleus, in close spatial and temporal synchrony with the apical expansion of the membrane bleb, to a height several microns above the initial nuclear surface. Previous studies have shown that nuclear deformations such as ‘nuclear blebs’, see for example Fig. 1g (red arrow), can result from uniform compressive forces applied along the apico-basal axis of cells, resulting in flattening of the nucleus and an increase in nuclear projected area^25^ (illustrated in Supplementary Fig. S1). The mean projected area of Cof/ADF depleted cells was increased relative to controls (Supplementary Fig. S1), as also observed previously^22^. However, nuclei in Cof/ADF depleted Hela cells were not flattened as would be expected in the context of global nuclear compression (Supplementary Fig. S1). Rather, the local displacement of the nucleus both laterally and apically to create bent extensions with large heads and thin bodies is suggestive of localized directed tensile forces that act to pull portions of the nucleus both outward and upward. Apical nuclear extensions were invariably found to be embedded in plasma membrane blebs and showed good correlation to plasma membrane bleb features such as size (Fig. 3b, Supplementary Figs S1 and S2). The majority of plasma membrane blebs do not contain nuclear extensions implying localized engagement between the plasma membrane and the nucleus. The morphologic and temporal coupling between apical nuclear extensions and plasma membrane blebs suggested the nucleus was dragged by linkage to the plasma membrane. Using optical tweezers we measured towing forces likely exerted on the nucleus, through coupling to plasma membrane bleb expansion, to be on the order of ~20–200 pN according to bleb size (Fig. 3c,d). While previous reports have highlighted the influence of compressive cytoskeletal forces to nuclear shape^14^,^15^,^25^, our results suggest that nuclear architecture is in fact susceptible to a variety of actomyosin induced stresses including tensile forces.

### ROCK-dependent actomyosin forces drive nuclear abnormalities following cofilin/ADF depletion

**Figure 4:**
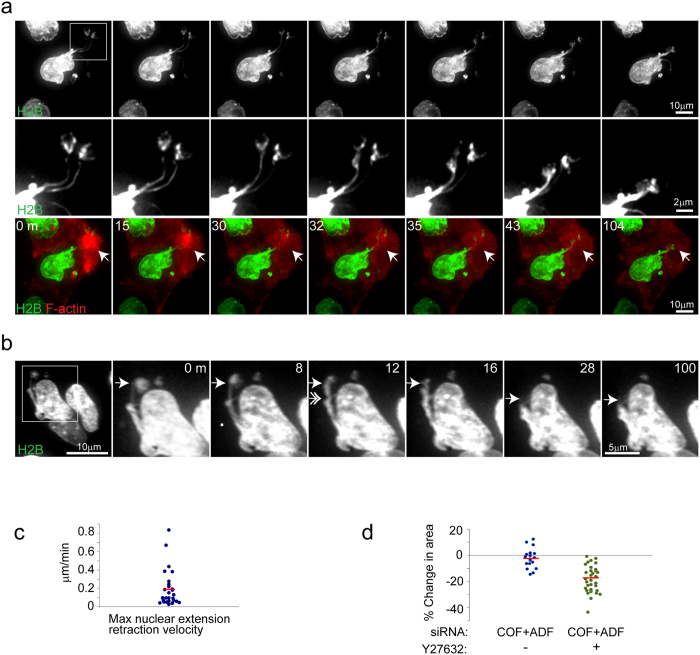
Nuclear extensions and F-actin aggregation are dependent upon ROCK activity in Cof/ADF depleted HeLa cells. (**a**) Time-lapse series of nuclear (GFP-H2B) and F-actin (RFP-Lifeact; red, merged images in bottom panels) alterations following treatment of COF + ADF depleted live HeLa cells with the ROCK inhibitor H1152. Arrow (bottom panels) shows disassembly of an abnormal F-actin aggregate following drug addition. Middle panels show magnification of boxed region (top panel, t = 0 m), depicting retraction of nuclear extensions. (**b**) Time series showing buckling behavior (double arrow) of a nuclear extension (arrow) during retraction, following Y27632 (Y276) treatment. Boxed region (first panel) is magnified in subsequent panels. (**c**) Quantification of nuclear extension retraction velocities following ROCK inhibition. Bar is mean value (n = 28). (**d**) Quantification of relative % change in nuclear projected area in untreated (n = 20) or Y27632 treated (n = 34) Cof/ADF siRNA depleted cells. Bars are mean values.

**Figure 5:**
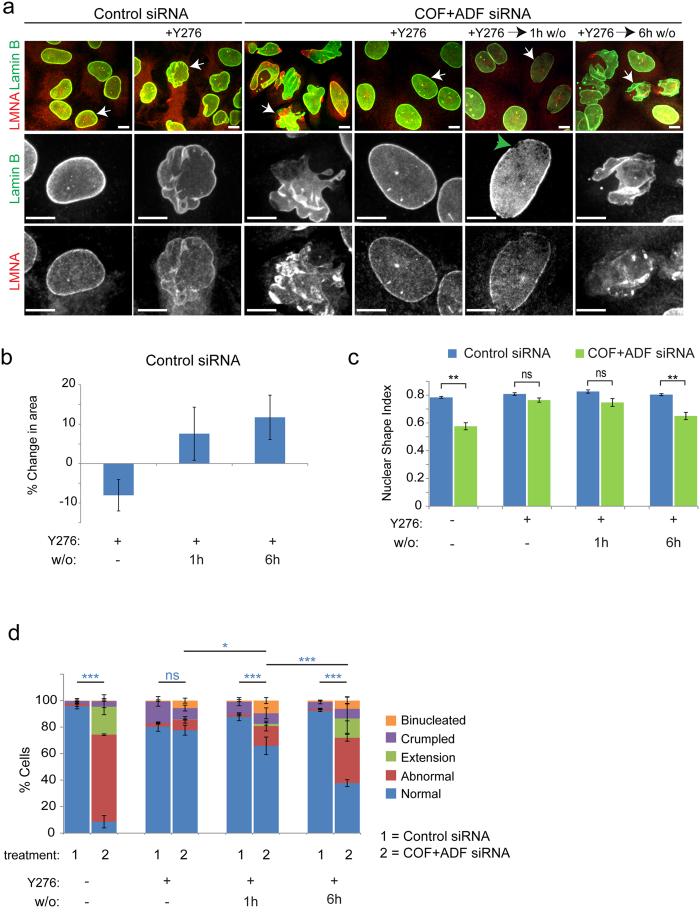
Nuclear architectural defects are temporally coupled to myosin-II activity in Cof/ADF deficient cells. (**a**) Representative immunofluorescence images of HeLa cells treated with siRNAs and the ROCK inhibitory drug Y27632 (Y276) as indicated. Cells were maintained under treatments up to 72 h or subjected to drug wash out (w/o) for the specified time periods, prior to fixation. White arrows depict cells shown in enlargements in lower panels. Green arrow illustrates cell with aberrant lamin B organization. Bar, 10 μm. (**b**) Quantification of nuclear projected areas for control cells treated with control siRNA and ROCK inhibitor Y27632 as specified. Values are mean ± SD, n > 450/treatment. (**c** and **d**) Quantification of nuclear abnormalities and shape index. Values are mean ± SD, n > 450/treatment. **p < 0.01, Welch’s t-test (**c**); *p < 0.05, **p ≤ 0.01, ***p ≤ 0.001, one-way ANOVA and Tukey’s post hoc for tests between indicated colored groups (**d**); ns, not significant.

ROCK activity is sufficient to drive nuclear structural disarray (Fig. 2i,j), in accord with previous reports^26^,^27^. Hence, ROCK signaling may aid in increased myosin activation and nuclear abnormalities following Cof/ADF depletion. Acute pharmacological inhibition of ROCK with H1152 potently blocked membrane blebbing (not shown), caused disassembly of bleb associated aberrant F-actin accumulations (Fig. 4a), induced nuclear area contraction (Figs 4d and 5b) and led to the recoil of nuclear extensions (Fig. 4a–c). The recoil behavior of nuclear extensions, including apparent buckling during recoil (Fig. 4b) indicates elastic properties for the nucleus. On the other hand, the fact that these extensions retracted slowly over a period of many minutes relative to their rapid formation (Fig. 3b) is consonant with overall viscoelastic features. Moreover, retracted extensions were not fully restored to normal nuclear shape over an hour after drug treatment suggesting some amount of plasticity to the nucleus. Together, these data support the prospect that nuclear extensions in HeLa cells are initiated and maintained by active ROCK-myosin-II dependent intracellular tensile forces pulling on a nucleus with evident viscoelastic properties, mechanical features consistent with the results of previous studies^28^.

Continuous ROCK inhibition by treatment of HeLa cells with Y27632 beginning at 24 h post siRNA treatments up until 72 h later, the point at which phenotypic assessments were generally conducted, efficiently reversed all types of nuclear structural defects in Cof/ADF silenced cells (Fig. 5a,c,d). ROCK inhibition of control cells also led to crumpling of the nuclear envelope and reduced nuclear projected area, suggesting normal nuclear spherical morphology and size are partly dependent on myosin-II induced prestress (Fig. 5a,b,d). Importantly, prominent nuclear architectural abnormalities including formation of extensions and disruptions to the nuclear lamina were temporally induced upon wash out of the ROCK inhibitory drug from Cof/ADF depleted cells whose nuclear morphology was normalized by prior ROCK inhibition (Fig. 5a,c,d). Disruptions to the nuclear lamina, evident by peripheral lamin B discontinuities, were predominant at 1 h post drug wash out and preceded more pronounced shape aberrations observed by 6 h post drug wash out. Increased focal aggregation of LMNA but not lamin B (Fig. 1e,g and 5a), only after drug wash out, likely reflects localized LMNA recruitment in response to intracellular actomyosin forces as suggested by a recent report^29^. The relatively rapid induction of nuclear structural defects in Cof/ADF depleted cells, only following ROCK disinhibition, provides strong evidence that these defects are directly attributable to myosin-II generated forces.

**Figure 6:**
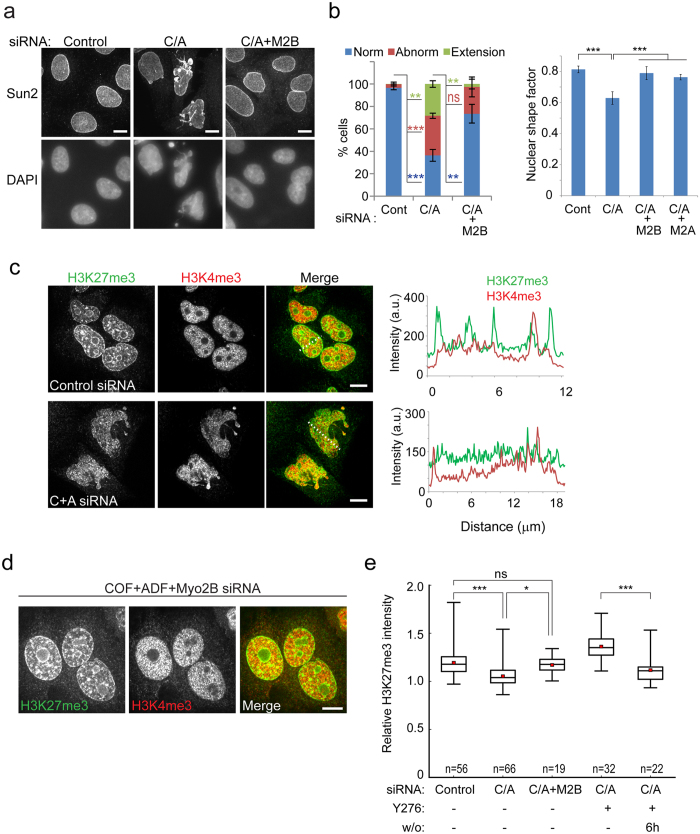
Myosin-II is necessary for abnormal nuclear morphologies and chromatin disorganization in Cof/ADF silenced cells. (**a**) Fluorescence images of nuclei, labeled for inner nuclear envelope protein Sun2 and by DAPI staining, from HeLa cells treated with siRNAs as indicated, showing rescue of nuclear morphological abnormalities by myosin-2B (M2B) codepletion with Cof/ADF (C/A). (**b**) Quantification of nuclear abnormalities and nuclear shape index. Values are mean ± SD, 300 ≤ n ≤ 1250/treatment. *p < 0.05, **p ≤ 0.01, *** p ≤ 0.001, ns, not significant, Welch’s t-test for comparison between indicated colored groups. (**c** and **d**) Confocal images of HeLa cells treated with siRNAs as indicated, fixed and dual immunostained at 72 h post treatment for H3K27me3 and H3K4me3. Graphs (**c**) show line fluorescence intensity profiles along the lines depicted in merged panels. (**e**) Quantification of peripheral relative to mean nuclear H3K27me3 intensity. Horizontal box lines show 25^th^, 50^th^ and 75^th^ percentile. Red square shows mean and whiskers show full range of data. Y276: 10 μM Y27632, w/o: drug wash out. *p < 0.05, **p ≤ 0.01, ***p ≤ 0.001, ns, not significant, one-way ANOVA and Tukey’s post hoc test.

To explicitly test the requirement for myosin-II in generation of the nuclear defects observed following Cof/ADF silencing, we co-silenced myosin-II along with Cof/ADF in HeLa cells. We previously showed that co-silencing of either of the two predominant myosin-II heavy chain isoforms expressed in our HeLa cells, myosin −2A and 2B, resulted in abrogation of increased actomyosin accumulations and features associated with increased myosin-II activity, such as membrane blebbing, in Cof/ADF depleted cells^21^. Here likewise, we found that co-silencing of either myosin-2A or myosin-2B isoforms effectively rescued nuclear morphological defects including formation of nuclear extensions and irregular shape (Fig. 6a,b and Supplementary Fig. S3). Nuclear abnormalities persisting following Cof/ADF and myosin-II codepletion consisted primarily of binculeated cells, a result anticipated based on known roles for these proteins in mitotic cell division. Similar to ROCK inhibition, myosin-II co-depletion also contributed to crumpling of the nuclear envelope in some cells, particularly in the case of myosin-2A co-silencing (Fig. 6a,b and Supplementary Fig. S3).

### Aberrant chromatin organization in cofilin/ADF depleted cells

The nuclear lamina contributes to chromatin organization, in part, through anchorage of heterochromatin at the nuclear periphery^30^,^31^. Since Cof/ADF depletion resulted in disruption of the nuclear lamina (Figs 1e,g and 2a) we examined whether heterochromatin organization was altered in Cof/ADF depleted cells. Chromatin organization was assessed by labeling of facultative heterochromatin, marked by histone H3 trimethylated at lysine 27, (H3K27me3) and euchromatin labeled by histone H3 trimethylated at lysine 4 (H3K4me3)^32^. Coincident with disrupted nuclear lamina structure in Cof/ADF depleted cells, we observed aberrant heterochromatin organization exemplified by reduced levels of both peripheral and perinucleolar H3K27me3 labeled DNA (Fig. 6c–e). Measurements of peripheral H3K27me3 relative to mean nuclear H3K27me3 intensity, from line profiles, demonstrated significant reductions to peripheral H3K27me3 in Cof/ADF depleted cells in contrast to control cells (Fig. 6e). Loss of peripheral H3K27me3 labeled heterochromatin was rescued by co-silencing of myosin-II or ROCK inhibition, indicating that this defect was also directly coupled to elevated myosin-II activity in Cof/ADF depleted cells (Fig. 6d,e).

### Disruption of the LINC complex and lamin A/C rescues nuclear abnormalities in cofilin/ADF silenced cells

**Figure 7:**
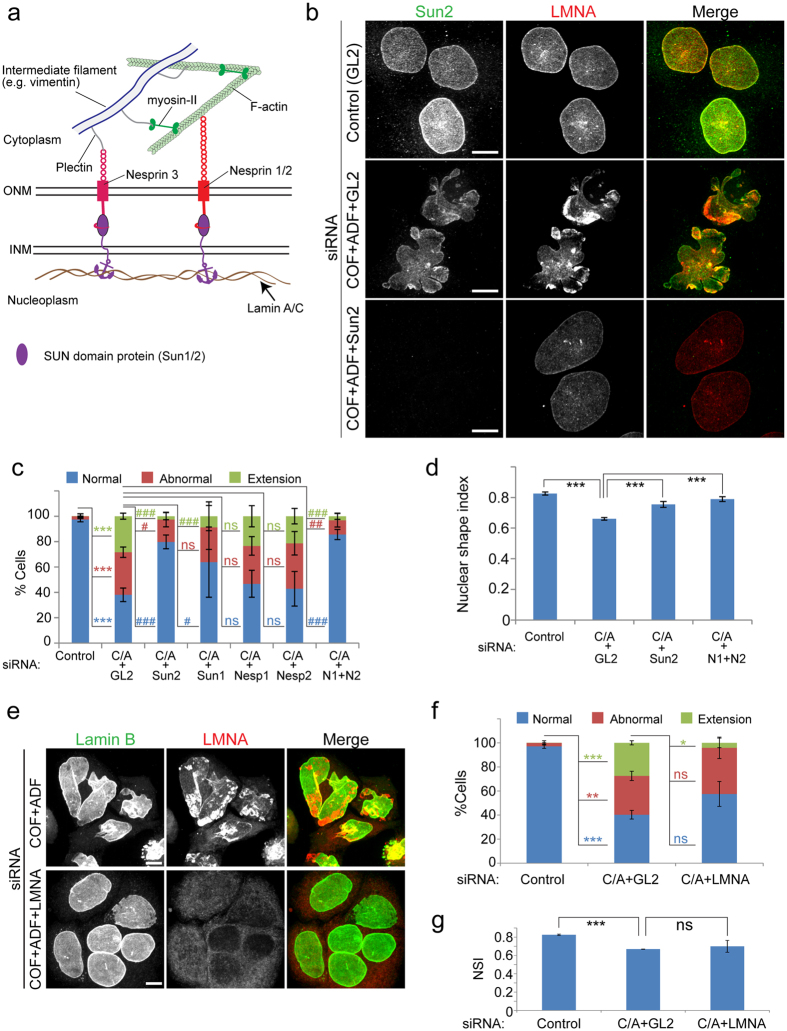
LINC complex nuclear envelope proteins are required for nuclear abnormalities following cofilin/ADF depletion. (**a**) Model illustration of LINC complex assembly across the nuclear envelope (ONM, Outer nuclear membrane; INM, inner nuclear membrane). (**b**) Representative immunofluorescence images of HeLa cells siRNA treated and immunolabeled as indicated, showing rescue of abnormal nuclear morphologies following Sun2 codepletion with Cof/ADF (C/A). GL2 is control siRNA to luciferase. (**c** and **d**) Quantification of nuclear morphological abnormalities (**c**) and nuclear shape index (**d**) in response to codepletion of the indicated LINC complex components with COF + ADF. Values are mean ± SD (n > 1000/treatment), using different siRNA oligonucleotides for each LINC protein (Nesp1/2, N1/2 – Nesprin-1 and Nesprin-2 respectively) in independent experiments. (**e**) Immunofluorescence images of lamin B and LMNA labeled HeLa cells, treated with siRNAs as shown. (**f** and **g**) Quantification of nuclear morphological abnormalities (**f**) and nuclear shape index (**g**, NSI) in response to LMNA and Cof/ADF codepletion. Values are mean ± SD, n > 300/treatment. Bars, 10 μm. *p < 0.05, **p ≤ 0.01, ***p ≤ 0.001, Welch’s t-test; ^#^p < 0.05, ^##^p ≤ 0.01, ^###^p ≤ 0.001, one-way ANOVA and Dunnett’s post hoc test; ns, not significant.

How are actomyosin forces transmitted to the nucleus in Cof/ADF depleted cells? The nucleus is mechanically coupled to the cellular cytoskeleton through nuclear transmembrane proteins that are anchored to the nuclear lamina, together known as the LINC complex^6^,^33^ (Fig. 7a). We therefore tested whether siRNA mediated co-silencing of LINC complex components could mitigate actomyosin force dependent structural abnormalities in Cof/ADF depleted cells. To ensure silencing specificity, for each LINC component we examined the effects of multiple siRNAs, targeting different sequences. Each tested siRNA reduced the nuclear protein levels of their respective target (Supplementary Fig. S4). Codepletion of either nesprin-1 or nesprin-2, outer nuclear membrane proteins which bind F-actin, did not significantly rescue abnormal nuclear morphologies including formation of nuclear extensions (Fig. 7c, p = 0.3 and 0.1, respectively for extension formation relative to Cof/ADF depletion alone). However, nuclear extensions and other morphological defects were markedly reduced upon co-silencing of both nesprin-1 and nesprin-2 with Cof/ADF (Fig. 7c,d). Inner nuclear membrane Sun-domain proteins bridge nesprins and tether the LINC complex to the nuclear lamina (Fig. 7a). Codepletion of Sun1 rescued the nuclear extension phenotype by over 60% (p = 0.02), however, total nuclear morphological defects were not significantly different from just COF + ADF depletion (Fig. 7c, p = 0.1); whereas codepletion of Sun2 with COF + ADF more efficiently rescued all nuclear morphological defects (Fig. 7c, p < 0.0001; Fig. 7b,d). Likewise, codepletion of LMNA with Cof/ADF rescued nuclear extensions (Fig. 7e,f, p = 0.004), however, these codepleted cells maintained some anomalies relative to shape and lamin B organization (Fig. 7e–g). Together, these results indicate a key role for LINC complex components in translating cytoplasmic actomyosin forces to establishment of nuclear deformities following Cof/ADF depletion. Our data also suggest partial redundancy amongst all LINC complex sub-families, including lamins, in mediating these features.

## Discussion

Our results assert an essential requirement for Cof/ADF-family proteins in the regulation of nuclear structure through control of the buildup of myosin-II intracellular contractile forces. Loss of canonical Cof/ADF F-actin disassembly function may be expected to result in F-actin stabilization and consequently to increased cellular F-actin content. Indeed, Kanellos *et al*.^22^ highlighted significance for F-actin accumulation for the generation of nuclear defects in Cof/ADF deficient cells. This conclusion was based on the observation that inhibition of F-actin nucleation mediated by Arp2/3, but not mDia1, rescued abnormal nuclear morphology and actomyosin accumulation. However, our combined results argue against simple increased F-actin polymerization as a critical factor to nuclear disorganization following Cof/ADF depletion.

Elevated F-actin assembly in close proximity to the nucleus, induced by active mDia1, failed to replicate the nuclear structural anomalies or signs of increased myosin-II contractile activity as observed in Cof/ADF depleted cells. It remains possible that a distinct geometry of actin filaments induced specifically by Arp2/3 may be required for nuclear deformation as recently implicated^34^. However, it is anticipated that such deforming filaments should persist following myosin-II inhibition, as shown^34^. Myosin-II co-silencing, for example, resulted in loss of stress fibers and other actomyosin structures in Cof/ADF silenced Hela cells (Supplementary Fig. S3 and ref. 21). Nevertheless, these cells maintained increased non-actomyosin F-actin structures at the cell cortex and displayed F-actin enrichment in membrane protrusions, commonly associated with Arp2/3 activity^35^, which became enhanced in size upon depletion of the two myosin-II isoforms expressed in these cells (Supplementary Fig. S3). Myosin-II inhibition led to effective rescue of nuclear defects in Cof/ADF depleted cells despite the ability of these cells to maintain elevated non-actomyosin actin assemblies. Together, these results lead us to conclude that F-actin stabilization is not by itself sufficient to induce nuclear dysmorphology in Cof/ADF deficient cells. F-actin assembly is a prerequisite to actomyosin assembly therefore it is to be expected that abolition of F-actin nucleation, which may rely on different nucleation factors dependent on cell type and cell status, would ultimately lead to reduced actomyosin assembly as observed by Kanellos *et al*.^22^.

We suggest that it is the specific increase in myosin-II association with F-actin, resultant from loss of Cof/ADF, as previously shown^21^, that principally drives nuclear structural aberrations. Increased actomyosin assembly and resultant elevated cytoskeletal tension in Cof/ADF depleted cells ostensibly creates a feedforward loop for increased myosin-II activation since intracellular forces can induce activation of RhoA GTPase^36^,^37^. This feedforward loop plausibly accounts for ROCK activation and increased myosin-II phospho-activation observed in Cof/ADF silenced cells (Fig. 2f–h and ref. 21).

While Kanellos *et al*.^22^ also showed that myosin-II activity was crucial for nuclear structural aberrations following loss of Cof/ADF, inhibition of ROCK activity did not rescue these deficits in mouse epidermal cells as we observed in our studies. Myosin-II phospho-activation is directed by different kinases and it is conceivable that other kinases such as myosin light chain kinase (MLCK) may be more critical for inducing light chain phosphorylation in mouse keratinocytes versus human HeLa cervical carcinoma cells.

We suggest that structural deformities resulting from Cof/ADF depletion are also not due to any direct structural necessity for Cof/ADF in the nucleus. We conclude this since nuclear aberrations in HeLa cells are effectively normalized by ROCK or myosin-II inhibition, indicating a requirement for Cof/ADF that impacts myosin-II regulation. Rather, our combined live and fixed cell analyses are consistent with nuclear anomalies arising in response to excessive cytoplasmic generated forces. For example, in live cells we observe rapid formation of nuclear extensions in association with non-apoptotic plasma membrane blebs and that bleb movement was directly translated to nuclear deformations. Nuclear extension formation coupled to plasma membrane blebbing, as observed in Cof/ADF depleted cells, may underlie an uncharacterized actomyosin dependent process leading to accumulation of nuclear fragments in blebs during apoptosis^38^.

The spring-like recoil of nuclear extensions following ROCK inhibition infers that certain nuclear deformities in Cof/ADF silenced cells are actively maintained by tensile actomyosin forces. Our measurements suggest that nuclei are likely to experience forces in the range 20–200 pN, predicated on mechanical coupling to the plasma membrane. This range of force is less than that estimated to be required for nuclear deformation as suggested by a recent report utilizing a micropipette aspiration procedure^39^. However, the force magnitude required for nuclear deformation will doubtlessly vary dependent on the mechanical properties of the nucleus itself. Nuclear deformities in Cof/ADF depleted cells are likely due to different types of mechanical forces but cannot be restricted only to compressive forces, as for example implicated previously^22^. By definition, a normal compressive force would be incompatible with upward extension of the nucleus, and without significant nuclear height reduction, as observed in Cof/ADF depleted Hela cells.

We observe that normalized nuclei in Cof/ADF silenced cells first exhibit disruption to the lamin B network prior to overt shape alterations, following myosin-II disinhibition (Fig. 5a). Disruption of the nuclear lamina by actomyosin forces may weaken the mechanical properties of the nucleus, inducing compliance and rendering it vulnerable to further force induced distortions.

Our data confirm a pivotal role for the LINC complex in facilitating force transduction between the cytoskeleton and the nucleus as LINC complex disruption rescued nuclear aberrations in Cof/ADF depleted cells. Beyond actomyosin accumulations surrounding some nuclear extensions in both Hela and RPE cells we did not observe, by diffraction-limited light microscopy, any distinct F-actin bundles linked directly to the nucleus, which could account for mechanical coupling to cytoskeletal tensile forces. Prior reports identified F-actin structures such as the perinuclear cap and transmembrane actin-associated (TAN) lines in association with cytoskeletal mediated nuclear shape changes and movement^14^,^40^. We detected F-actin bundles, congruent with a perinuclear cap structure, running over the apical nuclear surface in some cells such as RPE-1 (Supplementary Fig. S5). This structure was however not commonly observed in other cell types such as HeLa cells (Supplementary Fig. S5). Nevertheless, elegant electron microscopy studies leave no doubt that cytoplasmic cytoskeletal polymers link directly to the nucleus^41^,^42^. Moreover, myosin-II generated forces are likely to be transferred to the nucleus via LINC complex association with both F-actin and intermediate filaments as illustrated in Fig. 7a. This is the case since plectin, a cytolinker which binds nesprin-3 at the nuclear envelope^43^, links actomyosin to cytoplasmic intermediate filaments such as vimentin^44^,^45^. That the nucleus responds to mechanical feedback from an integrated cytoskeletal network of various interconnected filaments is supported by multiple previous studies that have demonstrated roles for both actomyosin and intermediate filaments in modulation of nuclear organization and movement^12^,^15^,^39^,^46^,^47^,^48^,^49^. Plectin, which interacts with plasma membrane bound factors such as integrins, spectrin and PIP2^50^, is also a candidate linker for the implied coupling between the plasma membrane and the nucleus, suggested by our data.

Loss of Cof/ADF induces disorganization of heterochromatin reminiscent of that associated with aging and diseases including cancer. Reduction to peripheral heterochromatin was observed within 6 h following myosin-II disinhibition in normalized Cof/ADF depleted cells. Our results suggest that the decline in peripheral heterochromatin may be consequential to disintegration of the nuclear lamina in response to actomyosin induced forces. Although lamin deficiency is known to cause peripheral heterochromatin loss^51^, it is also feasible that actomyosin forces may induce signaling which alter the activity of histone modifying enzymes^52^. These possibilities are not mutually exclusive. Regardless of precise mechanisms, our findings originate a premise by which deregulated cofilin and myosin-II activity may contribute to epigenetic nuclear modifications.

In conclusion, the results of this study establish cofilin/ADF proteins as crucial homeostatic regulators of normal interphase nuclear organization. Significantly, this function of cofilin/ADF is linked largely to modulation of myosin-II activity. Our study indicates that nuclear architecture is highly reliant on controlled intracellular actomyosin forces rather than general cytoplasmic F-actin organization. Furthermore, we establish that cofilin/ADF proteins, through control of actomyosin assembly, are pivotal in restricting the intracellular forces to which nuclei are subjected. Deregulated cofilin/ADF or ROCK activity has been tied to diverse human diseases ranging from Alzheimer’s to cancer^53^,^54^. Our results, for example, now suggest a mechanism for how altered cofilin and nucleo-cytoskeletal mechanocoupling, may contribute to perturbed nuclear lamina and chromatin organization recently identified for neuronal cells in Alzheimer’s disease models^55^. The findings herein thus provide a new avenue in which to examine the requirement of essential actin regulatory proteins in processes such as gene transcription and nuclear programming.

## Methods

### Cells and reagents

HeLa (Kyoto strain), RPE-1, Saos-2 and U2OS cells were grown in DMEM supplemented with 10% fetal bovine serum (Atlas Biologicals). Antibodies were rabbit, myosin-IIA, Sun1, Sun2, Nesprin-1, Nesprin-2, (Sigma), p-myosin light chain (Ser19 and Thr18/Ser19), (Cell Signaling); lamin A/C (sc-7292), lamin B (goat c-20) (Santa Cruz); H3K27me3, H3K4me3 (Abcam); GAPDH (Millipore); ADF/cofilin (1439, ref. 56).

### Transfections

Cells were transfected with 40–50 nM of siRNAs at the time of plating and again 24 h later with either Lipofecatamine RNAiMax or Lipofectamine 2000 (Invitrogen) using manufacturer’s protocols. Cells were cotransfected with a plasmid encoding ROCK-CA^26^, or infected with adenoviruses encoding mDia1CA (mouse p140mDia^57^, amino acids 535–1253).

### siRNA

siRNA oligonucleotides targeted to human ADF, cofilin, myosin-2A and myosin-2B were obtained as previously described^21^. Duplex siRNA oligonucleotides to human Sun1 #1-CGACACAGCTTTCCAAATA, Sun1 #2-CTGCAGGATGCTGTGACTCGA, Sun2 #1-TGCGAGAGCTGGAGAGCAA, Sun2 #2-CCGCATCGGGCTGGCAGACT, Sun2 #3–CCTATGGGCTGCAGACATT, Sun2 #4–CCTTAGAGCATGTGCCCAA, Nesprin-1 #1-CCAAAGACATTAAGGAAAT, Nesprin-1 #2-CAGGAGCTTCAGAGAGACATA, Nesprin-1 #3-CTGGAGTGGGATCACGACTAT, Nesprin-2 #1-GCAGAAATGTGTAGTATTA, Nesprin-2 #2–CCCGAGCATCACTACAAGCAA, Nesprin-2 #3–AAGGCTCATGTCACCGATCCA, lamin A/C #1–GCAAAGTGCGTGAGGAGTT, lamin A/C #2–CCGTGGAGGAGGTGGATGA, lamin A/C #3-CCAGGAGCTTCTGGACATCAA and a control siRNA targeted to luciferase (GL2) were obtained from Qiagen.

### Cell culture and microscopy

Fluorescent images were acquired with an Olympus IX81 spinning disk confocal (CSU22 head) microscope with either 100x/1.40 NA, 60x/1.42 NA or 40x/1.35 NA objectives. For live cell imaging cells plated on glass-bottom 35 mm dishes were housed in a stage incubator at 37 °C with CO_2_. Cells were co-transfected with siRNAs and/or plasmids encoding either Ruby or GFP-Lifeact^58^, GFP or RFP-H2B, GFP or RFP-CAAX (a gift of Gianluca Gallo). Phenotypic analyses of siRNA treated cells were at 48–72 h post initial siRNA treatment. For fixed cell microscopy, cells grown on glass coverslips were fixed in 4% formaldehyde in CBS buffer (10 mM MES pH 6.1, 138 mM KCl, 3 mM MgCl_2_, 2 mM EGTA, 0.32 M sucrose) + 0.4% Triton X-100 for 20 min at room temperature. Fluorescently labeled phalloidin and secondary antibodies were from Invitrogen. DNA was labeled by 4′,6-diamidino-2-phenylindole (DAPI) or To-Pro 3 (Invitrogen) staining. For drug studies live transfected cells were treated during imaging with 10 μM Y27632 (Sigma), 100 nM H1152 dihydrochloride (Santa Cruz) or DMSO. For Y27632 wash out experiments cells were treated with drug or PBS for controls, at 24 h post siRNA transfection and maintained in drug containing medium up until time of fixation or drug wash out. Confocal z-stacks (0.4–3 μm step size, 4–8 slices) of cells expressing fluorescently tagged proteins were acquired for live cell analyses.

### Optical Tweezers Bleb Force Spectroscopy

The optical tweezers setup was based on a 1070 nm continuous wave Nd:YAG laser (YLR-5-1070-LP, IPG Photonics, Oxford, MA) trapping beam, which was focused through a high NA objective (60X water immersion, 1.20NA; Olympus) of an IX-71 inverted microscope. Samples were moved using a piezoelectric stage (Nano-PDQ350, Mad City Labs Inc., Madison, WI) and imaged on a CCD camera (acA1300–30 gm, Basler AG, Ahrensburg, Germany) at a frame rate of 30 frames per second. The images were analyzed with a bead tracking algorithm in Labview (National Instruments, Austin, TX) based on cross-correlation that laterally localizes the bead center position with nanometer precision^59^. A Z-axis look-up-table (LUT) was generated using beads immobilized on glass, making axial localization of the trapped beads possible^60^. Trap calibration was performed through viscous drag force measurements applying triangular wave patterns to the piezoelectric stage and measuring the bead displacement in the trap^61^. The method employed provided a trap stiffness of 154 ± 19 pN/μm laterally and 67 ± 10 pN/μm axially (mean ± SD) for 1.7 μm polystyrene beads (Spherotech, Lake Forest, IL) when the laser power at the back aperture of the objective was 500 mW. In live cell measurements, a bead was trapped in the optical tweezers and positioned in contact with the cell membrane of an ADF/Cofilin depleted cell prior to bleb growth. Upon bleb formation at or near the bead contact site, the bead was displaced from the trap center. Tracking analysis recovered the bead displacement information, which was converted into force^62^.

### Image and data analysis

Quantitative results in all figures are from at least 3 separate experiments except for ROCK-CA expression in Fig. 1c which was the mean of two experiments. Persistent blebs were as defined^21^. DAPI stained nuclei were scored visually as abnormal in Figures 1c, 6b, 7c and 7f by the presence of multiple lobules, two or more invaginations, herniations or clear deviations from a smooth spherical morphology but without an extension. Nuclear abnormality and nuclear structural defects in other figures were assessed from nuclei stained for lamins A/C and/or lamin-B and additionally included nuclei with normal shape but with abnormalities to the nuclear lamina such as focal lamin aggregation and discontinuous peripheral lamin staining. Live cell fluorescence images were processed by background subtraction and a Gaussian filter. For cortical and bleb base p-MLC intensity quantification, 6–10 random 2 μm^2^ cortical regions/cell were measured. Peripheral and mean nuclear heterochromatin measurements were assessed from random line intensity profiles that traversed through the nucleus from the cytoplasm. Peripheral intensity was measured from peaks at the nuclear-cytoplasmic interface. The relative change in projected nuclear area was a measure of the percentage difference in the mean projected area of nuclei at each time point relative to the mean projected area of nuclei at the first imaging time point. Maximum nuclear extension retraction velocities were generated from kymographs. Generation of kymographs, morphometric measurements, quantification of fluorescence intensity and image processing were done using Metamorph, MATLAB, a Python package (Pyqtgraph; www.pyqtgraph.org), CellProfiler^63^ or Photoshop. Nuclear shape indexes were computed using CellProfiler^63^ or custom MATLAB scripts where nuclei were segmented by Canny edge detection or thresholding routines. Shape index was calculated by 4πArea/Perimeter^2^. Images were typically processed in CellProfiler with a pipeline as follows: 1) DAPI images of labeled nuclei along with other co-stained channels were loaded from the input module, 2) background subtraction was performed for each channel using modules Correct Illumination Calculate and Correct Illumination Apply, 3) segmentation of nuclei with module Identify Primary Objects (generally with a global Ridler Calvard thresholding procedure), 4) morphological opening to remove edge artifacts using module Morph, 5) fluorescence intensity and morphological features were computed using modules Measure Object Intensity and Measure Object Size Shape respectively. Segmentation binaries were saved for visual inspection and where necessary for manual editing and regeneration of measurements. Statistics were computed using Excel or R-Statistical software. A two-tailed Welch’s t-test was utilized or ANOVA followed by post hoc tests.

## Additional Information

**How to cite this article**: Wiggan, O. *et al*. Cofilin Regulates Nuclear Architecture through a Myosin-II Dependent Mechanotransduction Module. *Sci. Rep.* **7**, 40953; doi: 10.1038/srep40953 (2017).

**Publisher's note:** Springer Nature remains neutral with regard to jurisdictional claims in published maps and institutional affiliations.

## Supplementary Material

Supplementary Information
