# Supplementary material for: Cofilin Regulates Nuclear Architecture through a Myosin-II Dependent Mechanotransduction Module
Source: Sci Rep. 2017 Jan 19;7:40953. doi: 10.1038/srep40953 (PMC5244421; doi:10.1038/srep40953)
Supplement: Supplementary Information [file srep40953-s1.pdf]

Supplementary Information

Cofilin Regulates Nuclear Architecture through a Myosin-II Dependent Mechanotransduction Module

O'Neil Wiggan, Bryce Schroder, Diego Krapf, James R. Bamburg and Jennifer G. DeLuca

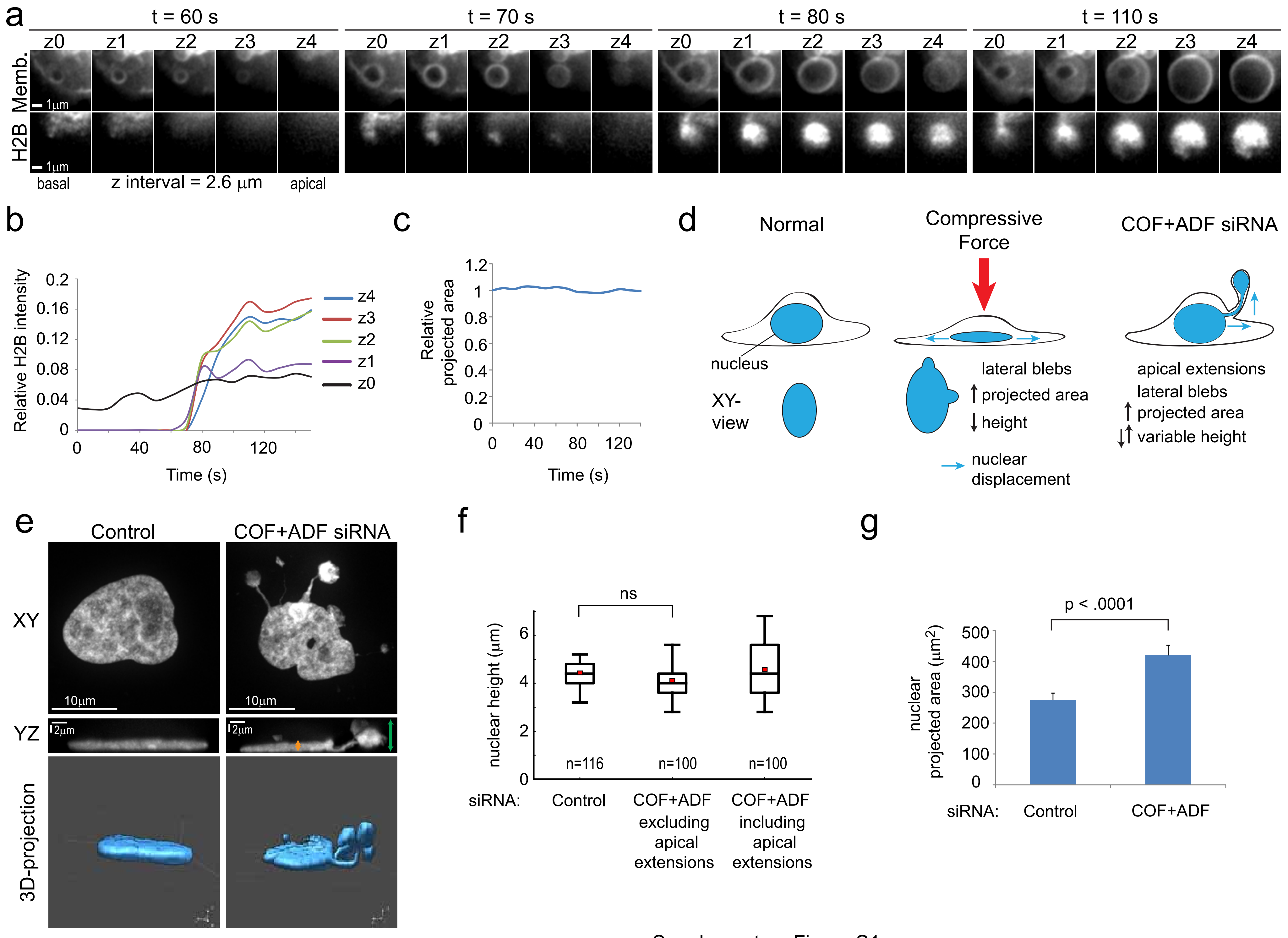

Supplementary Figure S1

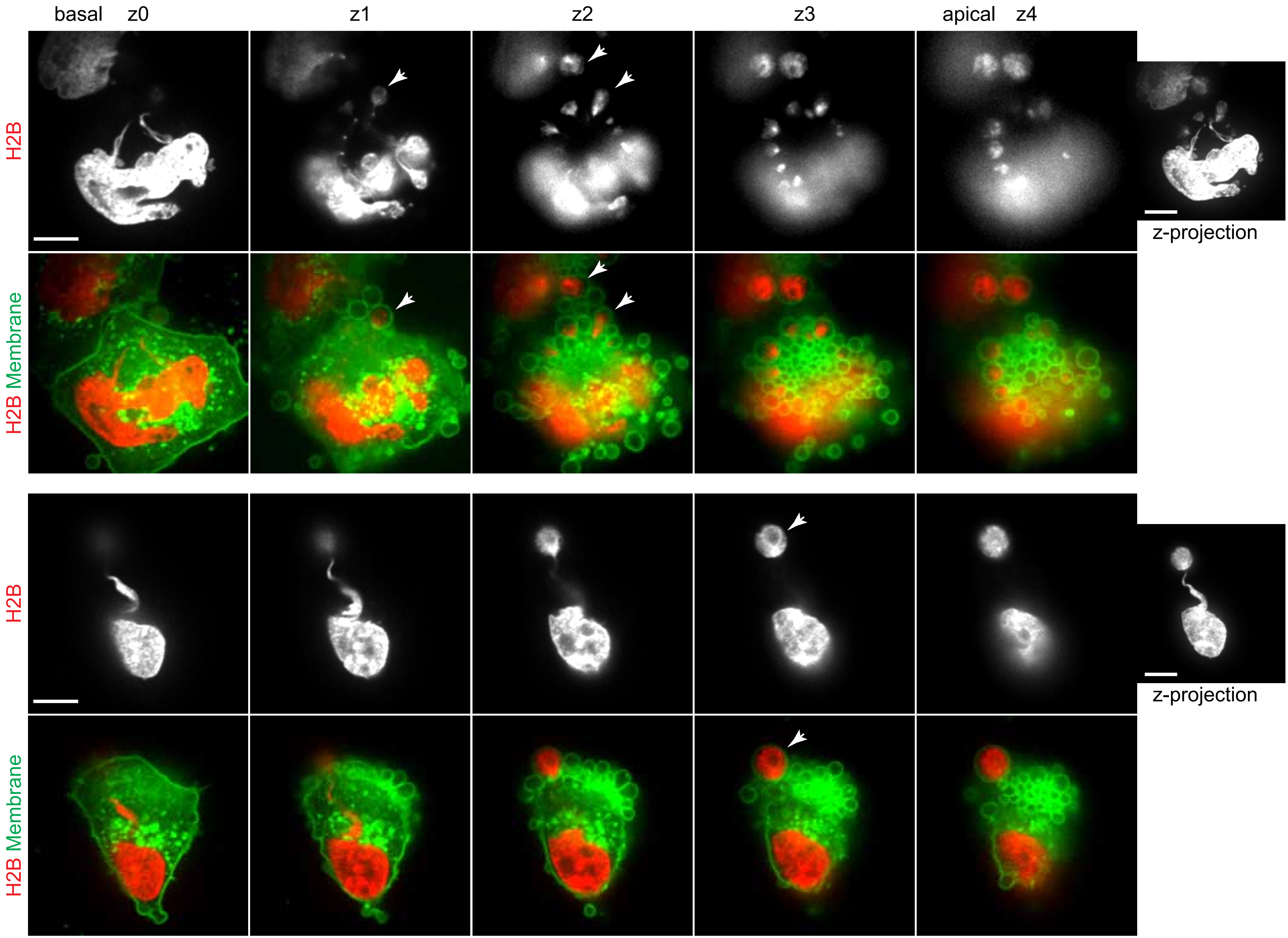

Supplementary Figure S2

Supplementary Figure S3

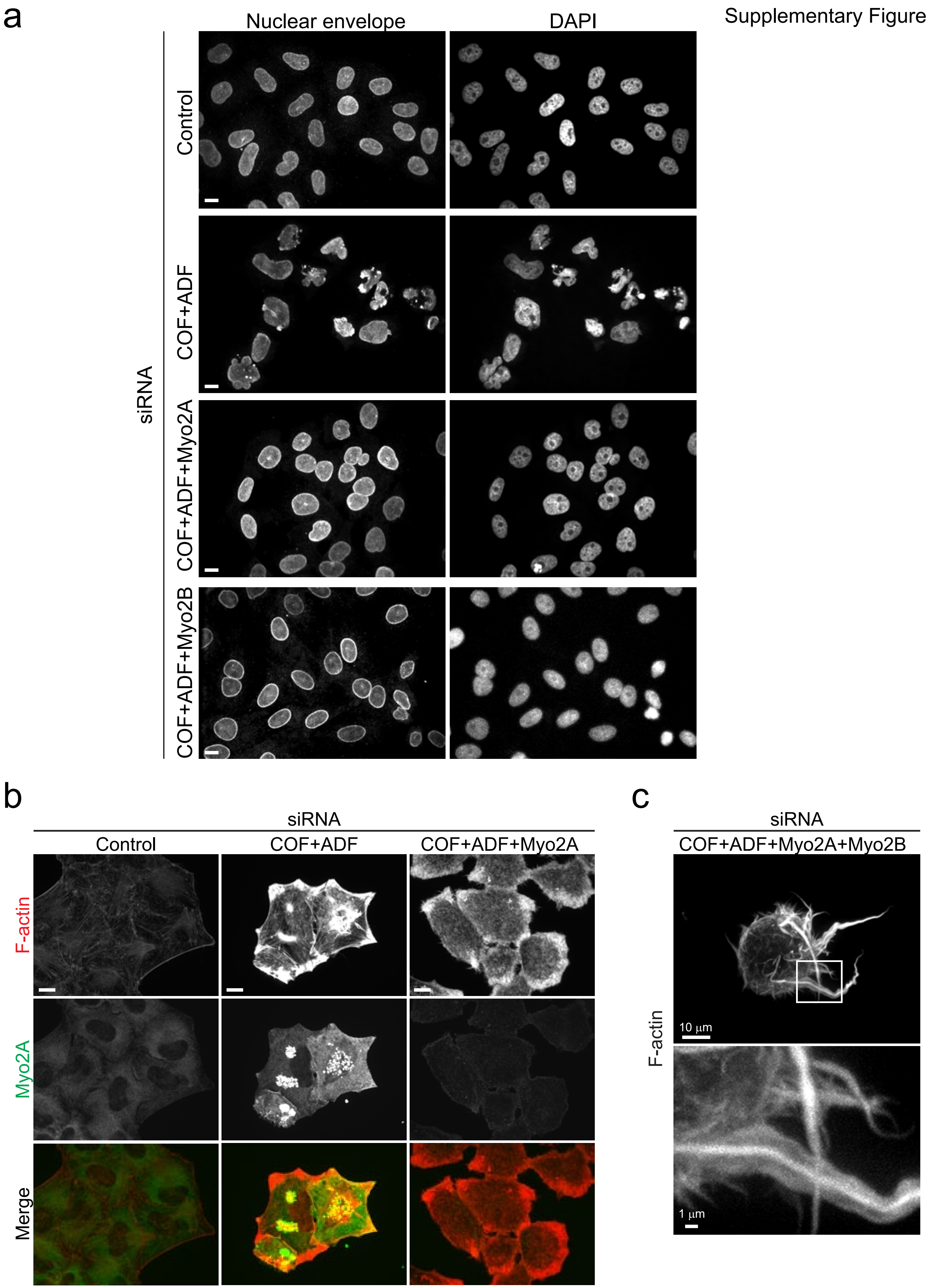

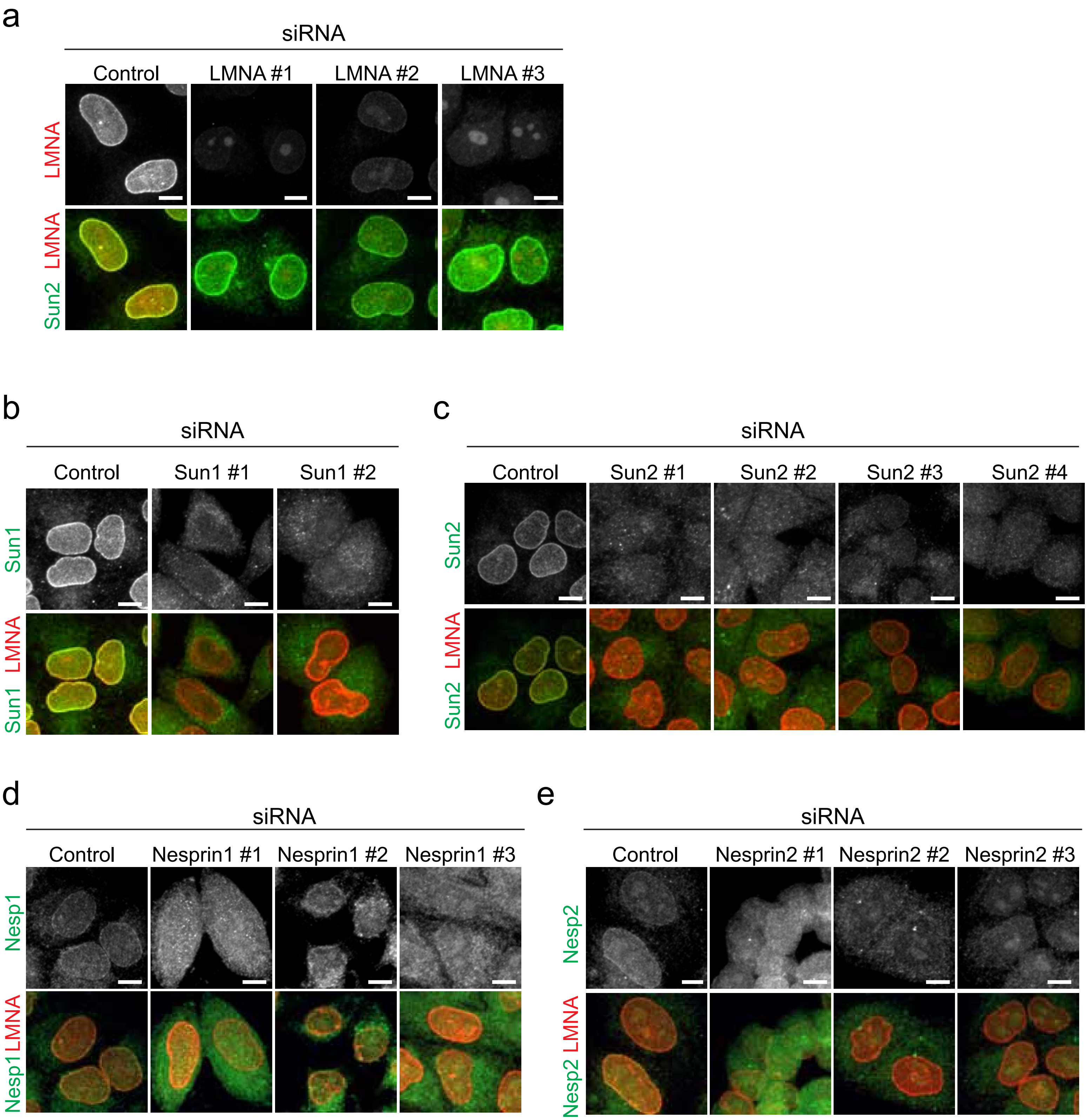

Supplementary Figure S4

a

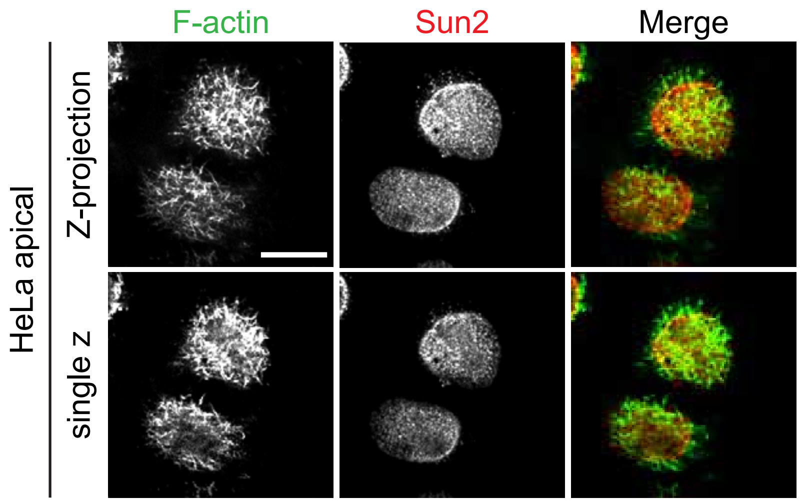

b

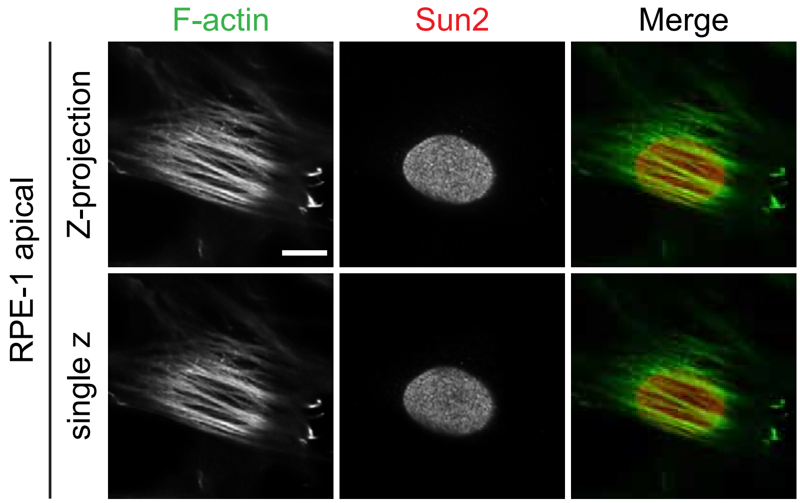

Supplementary Figure S5

a

Uncropped blots for Figure 1f

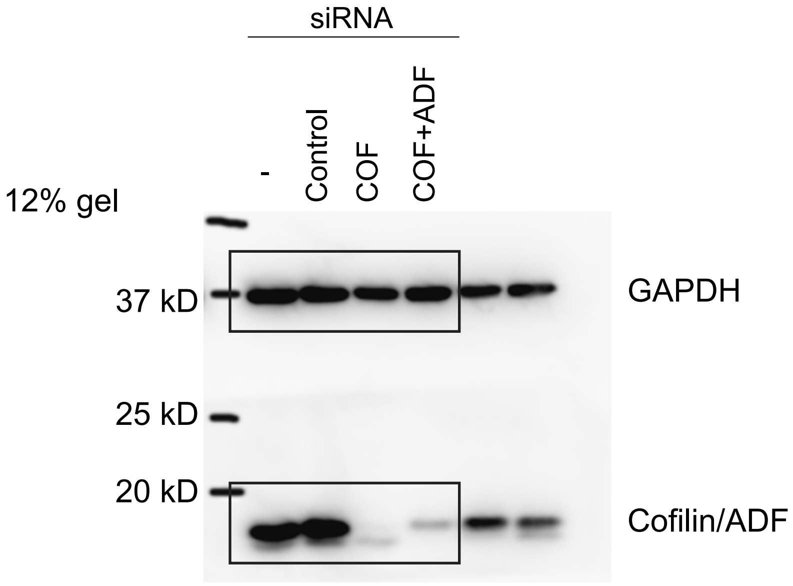

b

Uncropped blots for Figure 2h

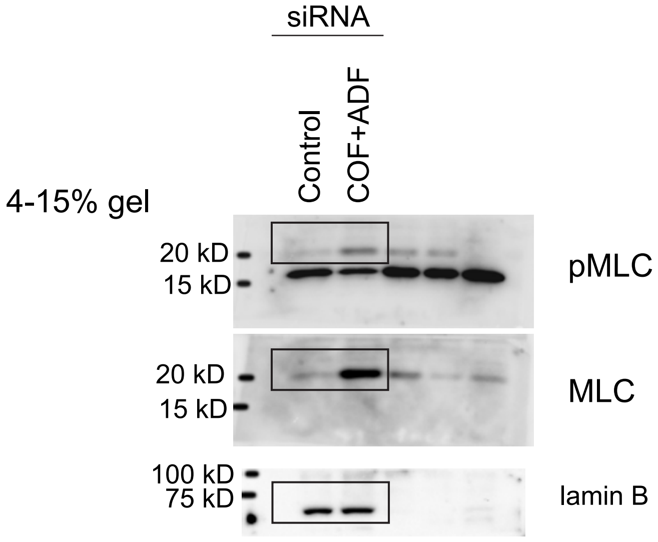

Supplementary Figure Legends

Supplementary Figure S1. Induction of apical nuclear extensions in conjunction with plasma membrane bleb expansion in cofilin/ADF depleted cells.

(a) Confocal time lapse z-series depicting formation an apical nuclear extension in tandem with the apical expansion of a plasma membrane (Memb.) bleb. Images are of region and cell depicted by box and arrows respectively in Fig. 3b. (b) Quantification of H2B fluorescence for boxed region, relative to time 0, at each z-plane shows temporal basal to apical rise of nuclear intensity. (c) Formation of the apical nuclear extension depicted in (a) was not associated with any substantial changes to projected area of that nucleus, as might be expected from episodic global nuclear compression. (d) Illustration of cell nuclear morphologies in response to different treatments. (e) Confocal XY (top), YZ (middle) and 3D-surface projections (bottom) of fixed DAPI stained HeLa cells, treated as indicated. Arrows illustrate the two different measurements made for nuclear height, quantified in (f). (g). Quantification of nuclear projected areas from fixed HeLa cells. Values are mean ± SD, n > 1000/treatment. P values from Welch's t-test.

Supplementary Figure S2. Apical nuclear extensions are embedded in plasma membrane blebs.

Confocal z-series of live HeLa cells where DNA was labelled by RFP-H2B and the plasma membrane by GFP-CAAX. Bars, 10 μm. Arrows illustrate encapsulation of nuclear extensions in apical blebs. Note also the correlation of size and morphologies between apical nuclear extensions and the membrane blebs that encapsulate them.

Supplementary Figure S3. Co-silencing of myosin-II isoforms rescues abnormal nuclear morphologies in Cof/ADF depleted cells.

(a, b and c) Confocal fluorescence images of fixed Hela cells at 72 h post siRNA treatments as indicated. The nuclear envelope is labeled by lamin A/C immunostaining for the first three rows

and by Sun2 for the last row of (a). Scale bars 10 μm, unless otherwise noted. Observe F-actin accumulation at the cortex and in the central region of enlarged filopodia-like protrusions (boxed region of c, magnified in lower panel) for cells co-depleted of cofilin, ADF and both myosin isoforms expressed in these HeLa cells.

Supplementary Figure S4. Silencing of nuclear envelope LINC complex proteins.

Immunofluorescence characterization of independent siRNAs for silencing of lamins A/C (a, LMNA), Sun1 (b), Sun2 (c), Nesprin1 (d) and Nesprin2 (e). Bottom panels show overlay with a non-targeted nuclear envelope protein as indicated. Cells were labeled at 72 h post siRNA treatment of HeLa cells. Bars, 10 μm.

Supplementary Figure S5. Examination of F-actin structures associated with the apical nuclear surface.

(a and b) Confocal immunofluorescence images illustrating F-actin organization above the nuclear surface, labeled by Sun2, of HeLa (a) and RPE1 cells (b). Shown are z-projections of all apical slices above the nucleus versus a single slice just above the nucleus. Bars, 10 μm.

Supplementary Figure S6. Full Western blots.

Full blots related to figure 1f (a) and to figure 2h (b). Cropped regions are boxed.
